# Starting at Go: Protein structure prediction succumbs to machine learning

**DOI:** 10.1073/pnas.2311128120

**Published:** 2023-09-21

**Authors:** James E. Rothman

**Affiliations:** ^a^Department of Cell Biology, Yale University, New Haven, CT 06520

## Abstract

This year’s Albert Lasker Basic Medical Research Award recognizes the invention of AlphaFold, a revolutionary advance in the history of protein research which for the first time offers the practical ability to accurately predict the three-dimensional arrangement of amino acids in the vast majority of proteins on a genomic scale on the basis of sequence alone [J. Jumper *et al.*, *Nature* **596**, 583–589 (2021) and K. Tunyasuvunakool *et al.,* *Nature* **596**, 590–596 (2021)]. This extraordinary achievement by Demis Hassabis and John Jumper and their coworkers at Google’s DeepMind and other collaborators was built on decades of experimental protein structure determination (structural biology) as well as the gradual development of multiple strategies incorporating biologically inspired statistical approaches. But when Jumper and Hassabis added a brew of innovative neural network-based machine learning approaches to the mix, the results were explosive. Realizing the half-century-old dream of predicting protein structure has already accelerated the pace and creativity of many areas of Chemistry, Biology, and Medicine.

## Proteins

Never has a scientific term been better chosen (or been more poorly understood at the time) than protein coined in 1838 by the Dutch chemist G. J. Mulder (1) [likely with prompting by Berzelius (2)] echoing the Greek prōteios (3) connoting “of the first order” a proposal based on similarities of chemical composition of still poorly defined extracts from animal and plants. Proteins remained ill-defined for the next six decades until it gradually (1900 to 1965) emerged that all proteins are polypeptide chains composed of 20 primary amino acids in a definite sequence prescribed by the genetic code, folding into unique shapes (conformations) that dictate their chemistry and therefore biological functions. Proteins are most certainly “of the first order” as little happens in the cell without them.

Understanding how proteins fold up into a unique conformation(s) thus became a central problem in biology by the 1960s with the prediction of protein structure expected to be a major benefit. It was recognized early on that each polypeptide chain can in theory assume an astronomical number of potential conformations, the vast majority of which would result in aggregation and precipitation (defining characteristics of almost all proteins when they are unfolded experimentally). Then in 1961, using the small secreted enzyme ribonuclease, Anfinsen (Nobel Prize, 1972) proved that the simplest possibility holds—that the sequence of amino acids, in itself, can determine the way the chain folds itself and that no additional genetic information is required in this process (4).

**Fig. 1.**
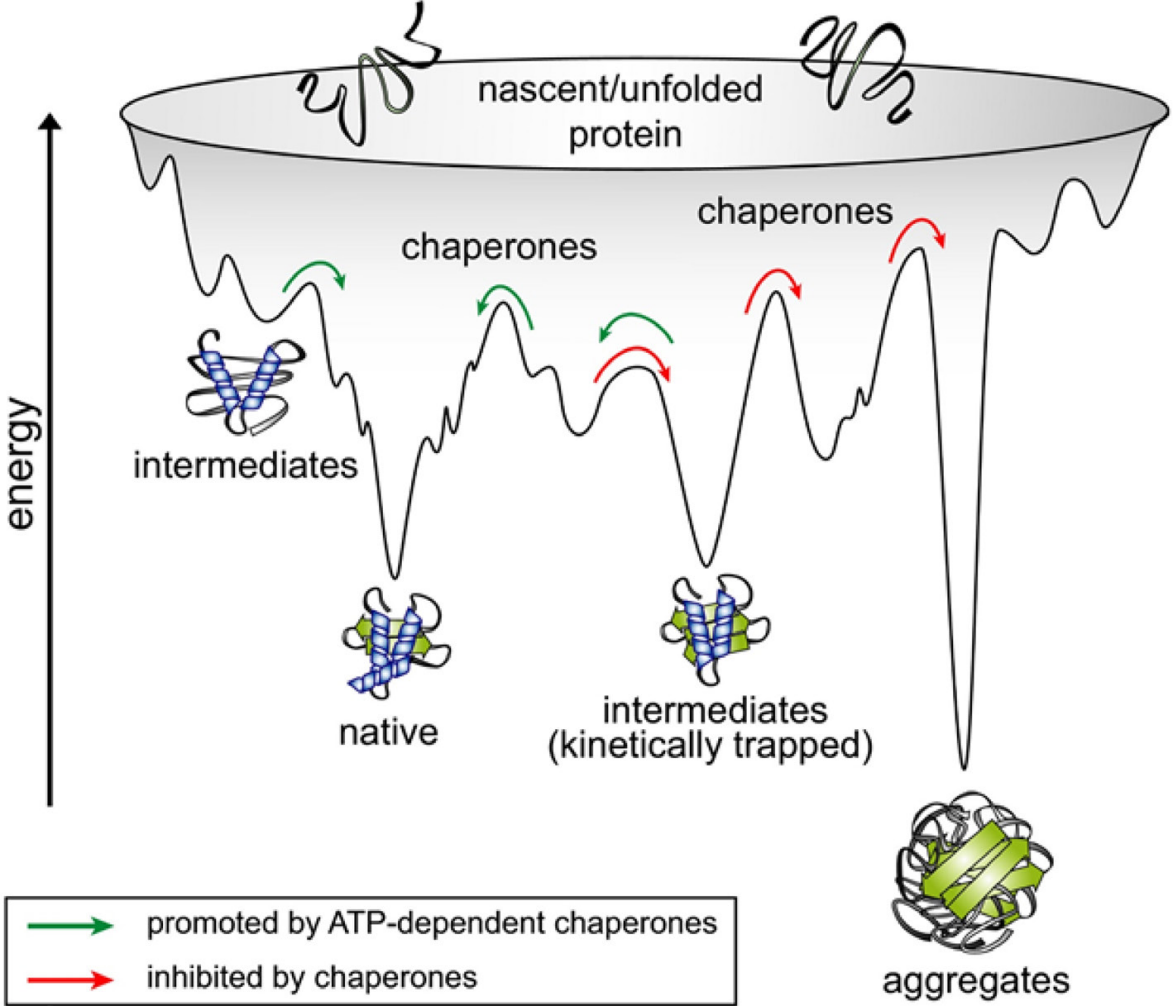
Molecular chaperones shape the energy landscape of protein folding. There is probably no one way to traverse the landscape; rather this is likely stochastic. During folding, proteins navigate a rugged, funnel-shaped potential free-energy surface en route to the native state. The accumulation of on- and off-pathway intermediates slows folding and entails the risk of misfolding into kinetically trapped states that are prone to form thermodynamically stable aggregates. Molecular chaperones inhibit aggregation, resolve kinetically trapped conformations, and provide kinetic assistance to folding by lowering free-energy barriers that separate folding intermediates from the native state. Image credit: From ref. 5, reprinted with permission from the American Association for the Advancement of Science (AAAS).

This pivotal experiment redefined protein folding as a problem in physics, the simple idea being that the folded state is the lowest accessible energy state of the polypeptide chain. But the extreme complexity of the corresponding energy landscapes, with many local energy wells to trap folding intermediates (Fig. 1) (5) made direct calculation and even simulation largely intractable in all but the simplest cases, even during a period when computing power was increasing exponentially.

By 1990, it became clear that protein folding in living cells is even more complex, requiring genes encoding molecular chaperones. In fact, the multidomain and multisubunit proteins, prevalent in highly evolved organisms, typically do not efficiently or rapidly refold in an Anfinsen-style experiment. This in no way contradicts Anfinsen’s core idea, that the final conformation is dictated solely by the physics of the polypeptide chain. But the discovery of molecular chaperones as catalysts of protein folding was a fundamental advance in cell biology (recognized by the Lasker Award in 2011 to F.-Ulrich Hartl and Arthur Horwich). Chaperone proteins (6) are in essence ATPases that channel energy from ATP hydrolysis to “kick” the folding polypeptide out of its energy well traps or avoiding such traps in the first place (Fig. 1) (5) all the while simultaneously shielding hydrophobic side chains to prevent aggregation (7, 8). In so doing, molecular chaperones catalyze folding overall by accelerating movement across the energy landscape.

## Predicting Protein Structure

As it gradually became evident that direct, physics-only approaches would ultimately fall short of atomic accuracy in the absence of revolutionary advances such as quantum computing, by around 2010 researchers had begun to turn to biologically inspired statistical work-arounds. One foundational idea (9) was that amino acids which are in physical contact in the folded structure, even if they are distant from each other in the amino acid sequence, will vary in evolution in correlated ways. These ideas are incorporated into the Multiple Sequence Alignments (MSAs) of homologous proteins that are now key to all predictive algorithms, including AlphaFold. Another foundational statistical concept involves structural Templates. Beyond homologous sequences, homologous three-dimensional structures can also serve as direct starting points for protein structure prediction. Template-based prediction was originally the dominant approach, but coevolutionary methods took over as information from DNA sequences grew explosively ahead of protein structures in the early 2000s. In the last 10 y, the tide has turned back to structures with the advent of cryoelectron microscope structure determination which avoid the bottleneck of crystallization.

The final and most important ingredient proved to be Machine Learning, enabling the information in MSAs and Templates, as well as other data, to be far more efficiently utilized (10). While information from MSAs and Templates can be utilized in separate pipelines, they can also be simultaneously utilized in interactive ways. This is essentially what “neural network” systems like AlphaFold do when they learn from the data the best way to combine them.

To assess progress objectively, the folding community established a biannual competition, the Critical Assessment of protein Structure Prediction (CASP) which measured the comparative accuracy of competing approaches for then-undisclosed experimentally solved structures. The DeepMind team (11) made its debut with AlphaFold (version 1) in the 13th edition of the meeting (CASP13 held in 2018) and did not go unnoticed (12), outperforming all 96 other competitors, though still falling far short of atomic-level prediction. Their algorithm generated the best structure for 25 out of 43 test proteins, compared with 3 out of 43 for the next best method, having a median accuracy of 6.6 Ås (the size of a Hydrogen atom is about 1 Å).

But Hassabis knew this was not good enough. According to Hassabis (quoted in ref. 13, “there was a difficult period after AlphaFold1. We first tried to push AlphaFold1 to the maximum. And we realized about six months after CASP13 that it was not going to reach the atomic accuracy we wanted to actually solve the problem and be useful to experimentalists and biologists. So, I made the decision that we needed to go back to the drawing board…. But for about six months to a year after that reset things got worse, not better. The AlphaFold2 system (the early one) was much worse than AlphaFold1. It can be very scary during the period where you seem to be going backward in terms of accuracy.”

John Jumper joined DeepMind in 2017 as a research scientist, in time to contribute a number of ideas to the CASP13/AlphaFold1 project, but soon introduced some new ideas that would mature into AlphaFold2. Hassabis was evidently impressed and promoted Jumper (July 2018) to lead AlphaFold2. Hassabis recognized the importance of Jumper’s interdisciplinary background in protein physics and machine learning. Critically, they shared the bold conviction that the prediction problem could be solved (i.e., accuracy could reach 1 Å on a genomic scale) in the face of the seemingly daunting odds suggested by AlphaFold2’s worsening performance. “Jumper was critical to the problem being solved,” he told me.

In the end, Jumper and Hassabis led their team to completely re-engineer the system including numerous innovations. The Nature paper describing the methods (14) is some 60 pages long and there are 32 different component algorithms. Although this August 2021 publication was a milestone, the key event actually occurred a year earlier when all entrants submitted their results to the CASP14 competition (May–July, 2020). When the results were unveiled (December 2020), the world of structural biology was stunned. AlphaFold2 (now mainly referred to simply as AlphaFold) had far outperformed all the competition, advancing prediction to atomic accuracy in a single step-change. AlphaFold now had a median polypeptide backbone accuracy of 0.96 Å. It predicted almost two-thirds of the target structures to an accuracy competitive with that of the best experimental results, ~1 Å deviation on the polypeptide chain backbone.

The outstanding performance of AlphaFold at CASP14, at that time not yet documented by publication, left the scientific community eager to learn details beyond the overall framework. Intrigued by the DeepMind still-unpublished results, David Baker (University of Washington), an internationally recognized leader in the related field of protein design, then explored network architectures that incorporated different combinations of the five main methodological advances described by Jumper and Hassabis at CASP14 (15). They incorporated related ideas including a three-track network (RosettaTTAFold) that produced structure predictions approaching those of AlphaFold in CASP14. Their paper was published in Science (15) within days of the Jumper-Hassabis publication in Nature (14) providing both valuable confirmation of the robustness of the DeepMind approach as well as a second algorithm for the community.

## How Does AlphaFold Work?

**Fig. 2.**
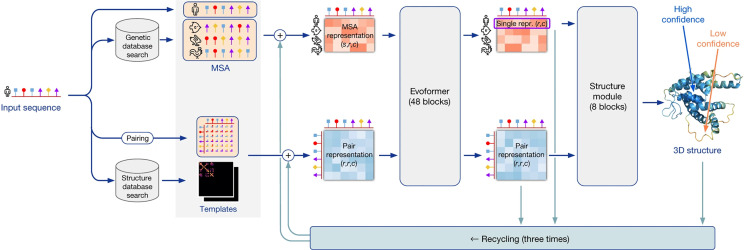
Model architecture of AlphaFold2. Arrows show the information flow among the various components. Array shapes are shown in parentheses with *s*, number of sequences; *r*, number of residues; and *c*, number of channels. Image credit: Reprinted from ref. 14, which is licensed under CC BY 4.0.

Even though the AlphaFold neural network was designed by humans, like the human brain, we may never fully understand it. AlphaFold is a product of engineering, and as such (unlike a biological brain), its internal anatomy is fully and explicitly known (Fig. 2). Understanding exactly how it works i.e., its internal dynamics, then becomes a scientific problem in and of itself. In neuroscience the classic approach is ablation, literally removing a part of the brain, or more subtly severing a connection, or even more subtly these days introducing an optogenetic lesion at the cellular level, or in the case of humans observing the functional deficits that result from lesions accompanying for example strokes. Jumper and Hassabis and their team carried out systematic computational ablation studies (14) that explicitly demonstrate that a variety of different mechanisms contribute in a way that largely sums up to the whole.

A basic mechanism in the design is a machine-learning artificial neural network that captures both long-range and short-range interactions, differing from previous algorithms that commonly considered only local or pairwise interactions, achieving this in part by taking into account fundamental aspects of polypeptide chain geometry. The use of iterative refinement similar to how the brain is thought to work is another central concept. Other key features are a new architecture to jointly embed MSAs and pairwise features; a new output representation and associated loss that enable accurate end-to-end prediction; use of intermediate losses to achieve iterative refinement; and novel training procedures involving self-distillation.

The network has two main stages (Fig. 2). First, the trunk of the network processes inputs through repeated layers of a novel network block termed Evoformer that ultimately represents processed MSAs and residue pairs and contains attention-based components. The ablation studies indicate that a concrete structural hypothesis arises within Evoformer and is continuously updated. Jumper and Hassabis explain in their paper (14) that key innovations in Evoformer include new mechanisms to exchange information within the MSA and pair representations that enable direct reasoning about spatial and evolutionary relationships. These updates essentially create trajectories along which the predicted structure converges on the correct solution and can be viewed as analogous to “moves” in explicit Molecular Dynamic or Monte Carlo simulations.

Second, the trunk is followed by the structure module that produces 3D structures in the form of coordinates for each residue. With each iteration, these rapidly develop and refine a highly accurate protein structure with atomic details. Key innovations include breaking up the chain structure to allow simultaneous refinement of all parts of the structure, perhaps providing a mechanism to avoid the multiple energy traps that carpet the landscape (Fig. 1) (5). Numerous other algorithms are introduced as well. Exact enforcement of peptide bond geometry is only introduced in the postprediction relaxation using an established (Amber) force field. Several recent papers provide accessible descriptions of the architecture and mechanism of the AlphaFold network (16–19).

The network was first trained from the open-access Protein Data Bank (20) (PDB), essentially the accumulated efforts of several generations of structural biologists. The PDB currently consists of ~200,000 unique protein structures and associated sequences, mainly from X-ray crystallography and more recently cryoelectron microscopy. The importance of this historical investment in structural biology cannot be overstated. Then, the trained network was used to predict the structure of ~350,000 diverse sequences from another important public library (Uniclust30) (21). Finally, a fresh AlphaFold network was trained from scratch using a mixture of PDB data and the dataset of predicted structures as the training data. This self-distillation procedure encourages the network to make use of sequence data that are unaccompanied by structures and was observed to improve accuracy overall.

As pointed out earlier, real proteins generally require molecular chaperones to access their lowest energy/folded states efficiently and without aggregation. As catalysts, chaperones accelerate the physical moves and thus the actual folding trajectory across the energy landscape. This juxtaposition strikes me as interesting because it suggests that AlphaFold somehow achieves the same end computationally. Perhaps there is an algorithmic equivalent of chaperones not explicitly identified as such that helps accelerate the computational excursion. As mentioned above, chaperones inject free energy (the equivalent in computation of noise) to produce local disorder that fluidizes movement across the landscape and do so in a very general way—a mere handful of chaperone species functions for a genome-worth of proteins.

## Starting with Go

Hassabis and Jumper’s success illustrates the creative energy that is unleashed when two fields fuse, in this case, machine learning with protein structure prediction. Demis Hassabis was born in London, England, of Greek Cypriot and Singaporean Chinese ancestry and was an official child prodigy, soon playing adults as a recognized chess master at 13. At 17, he joined Bullfrog productions where he designed games such as the bestseller, Theme Park. He then attended the University of Cambridge where he studied computer science and mathematics, and where he first heard about the protein folding problem from biology student friends. After graduating (1997), he joined Lionhead Studios and then founded his own successful company, Elixir Studios. Seemingly a nonlinear trajectory, it was anything but in Hassabis’ mind (13). “I’ve been working on and thinking about general AI for my entire career, even back at university. I tend to note down scientific problems I think one day could be amenable… and protein folding was right up there.” Yet he recognized that he still needed to learn how to approach science professionally (“the design of good control experiments and so on,” he told me), so he trained in cognitive science at University College London (PhD 2009). Following this, Hassabis founded DeepMind (acquired by Google in 2014) where he and colleagues initially focused on creating learning algorithms to master games, culminating in DeepMind’s AlphaGo stunning defeat of the legendary Go player Lee Sedol in 2016. “We pretty much started the [protein] project roughly the day we came back from the AlphaGo match in Seoul… We started off with games because it was more efficient to develop AI and test things out… But ultimately that was never the end goal (13).” Nearly two decades after Cambridge, Hassabis was ready to pull out his old notes.

John Jumper was born in Arkansas. His unconventional journey began relatively conventionally in physics and mathematics (Vanderbilt, BA 2007) followed by a year Marshall Scholarship to the University of Cambridge. Unsatisfied, he decided to take a break as a “quant” in finance and in 2008 applied to the storied firm DE Shaw in New York, founded by David Shaw, a computer scientist. As it happened, but unbeknownst to Jumper, until he applied, Shaw had returned from investing to basic research and was specially designing computers to tackle protein folding and drug discovery at D.E. Shaw Research, and he asked Jumper to join his research team. After 3 y of working on the forefront of computation, Jumper was both committed to the problem but also well aware that he would likely never again have available such awesome computational capacity. When he returned to graduate school, now at the University of Chicago (PhD in Chemistry 2017), he continued his research on protein structure prediction, but now focused on developing computation-sparing machine learning methods. Hearing rumors that DeepMind was getting into folding after Go, he managed to get his CV to Hassabis, and the duo recognized by this year’s Albert Lasker Basic Medical Research Award started working together in 2017.

## Kepler not Newton

Even as we laud the invention of the first technology that can accurately predict protein structures from gene sequences on a genomic scale, it is somehow also a bit disquieting to many scientists that we haven’t extracted generalizable principles as expected for traditional scientific explanations (22). With Kepler’s laws, we could accurately predict the orbits of planets, but the full power of Kepler’s technology was only unlocked when Newton extracted the principles of classical mechanics from them. There are many examples of this. Röntgen’s X-rays, which were discovered in 1895 and received the first Nobel Prize in Physics in 1901, had a spectacular impact on medicine long before they could be finally understood from quantum mechanics. In the fullness of time, we can hope that similarly deep insights and new principles (23) will emerge concerning how proteins actually fold up (as distinct from the shape they end up in) (24).

## Impact

No doubt the current version of AlphaFold is just the beginning (25, 26). Already it is being improved upon to include multisubunit protein complexes. As with any revolutionary and robust technological concept, it will attract open innovation and evolve in unpredictable ways to touch every corner of biological science. Literally every lab I know is using AlphaFold today in ways that are often highly speculative and imaginative. Even the inevitable failures in these countless probing expeditions will catalyze improvements on many fronts. The greatest long-term impact of AlphaFold, however, may be to signal that AI is about to become an accepted, reliable, and useful method for discovery in biology. Today’s scientists were mostly trained to use models but not really to trust them, especially if they do not understand them explicitly. Are we ready to trust first and (only occasionally) verify?

## Data Availability

All study data are included in the main text.
